# The Influence of Teaching Approach on Students’ Conceptual Learning in Physics

**DOI:** 10.3389/fpsyg.2018.02474

**Published:** 2018-12-05

**Authors:** Lucia Bigozzi, Christian Tarchi, Carlo Fiorentini, Paola Falsini, Federica Stefanelli

**Affiliations:** ^1^Department of Education and Psychology, University of Florence, Florence, Italy; ^2^Centro di Iniziativa Democratica Insegnanti (Center of Teachers’ Democratic Initiative), Florence, Italy

**Keywords:** physics, conceptual learning, critical thinking, teaching approach, epistemic beliefs

## Abstract

Physics is fundamental to secure future needs for scientific and technological competence (Angell et al., 2004), but many countries experience a drop in students’ performances in international assessments (Organisation for Economic Co-operation Development [OECD], 2018), as well as in rates of enrolment in undergraduate programs in scientific disciplines (STEM). Socio-constructivist theories have produced a reforming movement in several educational systems, in particular in the area of sciences, but teacher often consider them an idealistic view of education and do not consider themselves metacognitively competent enough to foster thinking in the classroom. In this study, we investigated the efficacy of different teaching methods on high-school students’ conceptual knowledge of physics, after the effect of science-related beliefs and critical thinking skills was controlled. We adopted a mixed-method with sequential design, in which quantitative and qualitative data flow are inter-mixed. In specific, we interviewed four high school physics teachers to identify teaching approaches (qualitative approach) and compared them in terms of efficacy on students’ performances (quantitative approach). Four teachers and 77 10th grade students participated. Teachers were interviewed during the school years and asked questions about their teaching experience, their teaching approach (Kang and Wallace, 2005) and their epistemic beliefs (Tsai, 2002). Students performances in Science-related beliefs (Conley et al., 2004), critical thinking (Cornell Critical Thinking Test Level X, Millman et al., 2005), and conceptual knowledge in physics (The Force and Motion Conceptual Evaluation, Ramlo, 2002) were evaluated twice, at the beginning and at the end of the school year. The independent-sample *t*-tests on pre-test variables did not reveal any statistically significant difference between groups. Results from the complex samples GLM revealed statistically significant differences on post-test scores in conceptual knowledge in physics, after the effect of covariates was controlled. Overall, the study contributes to our understanding on current teaching practices in school, and their effect on students’ conceptual understanding of physics concepts.

## Introduction

In several countries there is great concern about students’ performances in science. International assessments, such as PISA (Program for International Student Assessment) or TIMSS (Trends in International Mathematics and Science Study) have revealed a high percentage of underachieving students, and a low percentage of excellent performance in science. According to PISA, although students express interest in science topics and recognize that science plays an important role in the world, their performances are not excellent, and greatly depend on how science is taught in their schools (Organisation for Economic Co-operation Development [OECD], 2018). According to TIMSS, no countries show a significant increase in performances in Physics from 1995 to 2015 in students, and only a small percentage reach the high benchmark (Stephens et al., 2016). Not surprisingly, few students enroll in undergraduate programs in scientific disciplines (STEM) compared to other domains, and even adults fail at understanding science-related topics when they are brought to their attention, affecting their decision-making processes.

Thus, it is crucial for research to focus on the influence of how science is taught in schools on students’ conceptual learning of science (Bigozzi et al., 2002). In this study, we will focus on Physics, and in specific on high school students’ conceptual learning of force and motion. There are several reasons why high school students struggle in learning physics concepts. In specific, in this study, we investigated the effect of students’ pre-instructional conceptions of physics, science-related beliefs, and critical thinking.

### Conceptual Understanding of Physics in High School

Physics was one of the first areas in which students’ pre-instructional conceptions were studied (e.g., McCloskey, 1983; Aretz et al., 2016). Students’ pre-instructional conceptions that are deeply rooted in daily life experiences have been defined in several ways (e.g., misconceptions, alternative conceptions, intuitive conceptions, naive conceptions, and the like), and there is a plethora of studies showing that they impair their conceptual understanding of science topics (Vygotsky, 1978; Ramlo, 2008; Bigozzi et al., 2011, 2014; Vosniadou, 2013). Rather than being blank slates, students begin physics with a well-established set of theories grounded on their common-sense beliefs about how the physical world works (Hestenes et al., 1992). If instruction does not take students’ pre-instructional conceptions into consideration, it will be almost totally ineffective (Hestenes et al., 1992). Of notice is that conceptual change is domain-specific, that is new information obtained through experience and/or instruction can lead to a specific restructuring in a delimited area of our knowledge. Two fundamental types of conceptual change have been hypothesized: weak and radical restructuring (Carey, 1985). In weak structuring, new information is integrated in pre-existing schemes, causing an increase in the relationships among concepts, but without altering the fundamental attributes; in radical restructuring, new information determines a change in the structure of the individual’s concepts and relationships between concepts. For what concerns the topic of force and motion, prior studies have established that common-sense beliefs are incompatible with Newtonian concepts (Hestenes et al., 1992), calling for radical restructuring as an aim of instruction. An example of radical restructuring in the physical domain of force and motion would be a shift from thinking of force as an entity to thinking of it as even a process (Ramlo, 2002). In the next paragraphs, we will discuss two individual difference variables (i.e., science-related beliefs and critical thinking) and a contextual variable (teaching approach) that have been found to be associated with students’ conceptual learning in physics.

### Science-Related Beliefs

Students’ epistemic beliefs, that is beliefs about the nature of knowledge and knowing, are receiving increased research interest in several domain of knowledge (see Greene et al. (2016)). Since Schommer’s (1990) seminal studies, students’ epistemic beliefs have been repeatedly associated with conceptual learning in science. Hofer and Pintrich (1997) suggested that four dimensions represent students’ beliefs about the nature of knowledge and knowing. The former ones are reflected by the certainty and development dimensions: students vary in the degree to which they believe that there is always a right answer or, conversely, whether there may be more than one answer to complex problems; and in the degree to which they think that theories can evolve and change or not. Students’ beliefs about the nature of knowing are reflected by the source and justification dimensions: students vary in the degree in which they believe whether knowledge originates from external authorities or is internally constructed; and in the ways in which they cite evidence and evaluate claims.

Several studies have found a relationship between epistemic beliefs and conceptual knowledge of physics (Stathopoulou and Vosniadou, 2007; Franco et al., 2012). For instance, Stathopoulou and Vosniadou (2007) studied this association in Greek secondary school students in two studies, and found that students with a high epistemological sophistication in physics reported a higher conceptual understanding of physics, as assessed by the Force and Motion Conceptual Evaluation instrument (Thornton and Sokoloff, 1998) than students with a low epistemological sophistication in physics. The authors concluded that sophisticated physics-related epistemological beliefs are necessary but not sufficient for conceptual understanding of physics. Franco et al. (2012) found that when undergraduate students’ epistemic beliefs were consistent with the knowledge representation of a physics text about Newtonian laws, they showed better learning than when their epistemic beliefs were inconsistent. Science-related beliefs and conceptual understanding of physics are associated, although the direction of this association is unclear. Mason et al. (2013) investigated the relationships between epistemic beliefs and achievement in science in three age groups (5th, 8th, and 11th graders). They found that for 11th graders the hypothesized model explained a smaller portion of variance in achievement in science as compared to the other age groups. Epistemic beliefs had a direct effect on knowledge in science, which in turn has a direct effect on achievement in science. Results from a developmental perspective suggested that in 11th grade only mastery goals directly influence domain knowledge. Thus, given the existence of an association between teaching approach and achievement goals (Urdan and Schoenfelder, 2006), it could be expected that in high school a constructivist learning environment may enhance students’ knowledge in science by inducing mastery goals, rather than performance goals (e.g., by creating a learning environment in which all ideas are equally useful, rather than asking students about their ideas with the purpose of correcting them).

### Critical Thinking

Critical thinking is considered as a necessary component of a 21st-century active citizenship that participates in a pluralistic and democratic society (Angeli and Valanides, 2009). For this reason, the development of this kind of thinking is considered the primary goal of science education (Tiruneh et al., 2017). Critical thinking is a type of reflective thinking, focused on deciding what we should believe or do (Ennis, 1989). A critical thinker needs the skills to identify what is implicit in reasoning and to judge if the basis of an inference is solid or not. According to Ennis (1989), it is possible to decide what to believe through different processes, namely induction, deduction, and value judgment. Each of these processes taps on several critical thinking skills: identifying the source of information, analyzing the credibility of information, comparing new information with prior knowledge, and drawing conclusions based on their critical thinking (Linn, 2000).

Initially, critical thinking was taught as a separate track from other subjects, whereas more recently efforts have been made to embed critical thinking skills within subject matter instruction (Niu et al., 2013; Tiruneh et al., 2014). The relationship between critical thinking and conceptual understanding in science is bidirectional: students need critical thinking skills to understand scientific concepts, but science learning might enhance their critical thinking skills, if the latter are targeted by the teacher and embedded in the curriculum. Successful teaching of CT skills in within the teaching of domain-specific knowledge should result in both, deeper conceptual understanding of the subject and development of critical thinking skills (Tiruneh et al., 2017). Miri et al. (2007) compared a group of high school students who were exposed to teaching strategies designed to enhance critical thinking in science classes to two other control groups, a science one and a non-science one. A mixed method research model was applied: critical thinking was measured at the beginning and at the end of the school year, and teaching strategies promoting critical thinking were identified through semi-structured interviews. According to the results, the experimental group showed a statistically significant improvement in critical thinking skills compared with the control groups. Teaching approach plays a fundamental role in mediating critical thinking improvement over time. The next paragraph will discuss the influence of teaching approach on conceptual understanding in science in general, and physics in specific.

### Teaching Approach

In the past half-century, literature on teaching has largely disputed whether students learn more in an unguided or minimally guided environment in which they must discover and construct knowledge, or, conversely, whether they should be provided with direct instructional guidance on discipline-specific concepts and procedures (Kirschner et al., 2006). The debate was initiated by the influence of constructivism on learning, which also produced several minimally guided approaches (e.g., discovery learning, inquiry learning, constructivist learning, and the like, Kirschner et al., 2006). Most of these approaches are implemented in science courses, in which students are asked to discover science laws and principles by acting as scientists (van Joolingen et al., 2005). However, there are several reasons why constructivism, interpreted in this way, is not widely used in educational systems. First, minimally guided environments may induce teachers to reduce the use of important aspects of learning, such as providing feedback. For instance, Zhang (2018) investigated the detrimental effect of withholding answers from students and found that students involved in hands-on activities with feedback provided achieved better science learning performances than students in hands-on only condition, with answers withheld, and students in the direct instruction condition. The author concluded that withholding answers during inquiry-based learning had hindered students’ understandings of concepts, development in reasoning skills, and ability to transfer knowledge to real-life situations. Some authors consider minimally guided constructivist approaches as theoretically incompatible with human cognitive architecture (Kirschner et al., 2006). For instance, working memory is limited, and problem-solving, a central component of constructivist approaches, places a huge load on working memory, which is not available to be used to learn (Kirschner et al., 2006). Moreover, assuming that the way an expert works in a domain is equivalent to the way in which a novice learns in the same domain might be a fundamental error, and research has consistently shown that guided instruction leads to better learning results than unguided instruction does (Kirschner et al., 2006). In a recent study, the beliefs of 87 science teacher about the differences between students’ experiments and scientific experiments were collected. According to the results, they considered all experimentation as a kind of science practice; however, these two types of experimentation are also characterized by differences. The three largest dimensions involved in students’ experimentation were pedagogical, procedural, and epistemic whereas, for scientific experiments, the major dimensions involved were procedural, epistemic, and materials (Wei and Li, 2017). These results demonstrate how the practice of experimentation should be substantially different when the students are involved in it. Indeed, in students-led experiments it is important that the teachers take into account the pedagogical dimension.

Rather than claiming that constructivist teaching approaches are ineffective, we propose to investigate how constructivist principles can be included in approaches in which the teacher is assigned a fundamental designing and managing role (e.g., guided instruction). As discussed earlier, constructivist theories recognize that students bring to science class pre-instructional conceptions on world phenomena derived from their everyday experiences, and they are not going to revise them if simply exposed to new theories, unless they are provided with reflective experiences (Boddy et al., 2003). Students’ pre-instructional concepts are often viewed by teachers as obstacles to science learning, but they may serve as resources if teachers increase their understanding about the range of possible ideas that students hold about science topics (Larkin, 2012). Socio-constructivist theories encourage teachers to focus more on inquiry (Mortimer and Scott, 2003) and student-centered instructional practices (Schneider et al., 2005). However, inquiry-based activities should be integrated with classroom talk (Mortimer and Scott, 2003). All science teachers recognize the importance of experimentation in teaching, but they often fail at introducing scientific discourse in their classes. Classroom discourse should not just be used as preparation for the experiment or as after-experiment analysis, but rather should be used to foster learning progression, to search for new knowledge or to answer new questions (Bereiter, 1994). Physics classrooms based on progressive discourse greatly increase students’ conceptual understanding of physics more than content-centered classrooms do (Bigozzi et al., 2014). Some studies have focused on science teachers’ use of laboratory in their teaching practices (Hofstein and Lunetta, 1982) and found that typically lab activities are used more as “a frill” rather than an integrated component of their course (Tobin, 1986; Kang and Wallace, 2005). The laboratory can be used in several ways: to verify a law, in this case, the laboratory is organized as a sequence of hands-on activities, in which students follow guidelines describing each step (how to mount the instrument, how to measure, and the like); or alternatively, students are let free in their inquiry, without any specific instruction; or, finally, the laboratory is perceived as a “break” from classroom lectures.

Finally, science teachers vary in the extent to which they aim at teaching content only or, conversely whether they integrate higher-order skills, such as critical thinking, in their program. Critical thinking is certainly a core component of teachers’ professional development, but only a very few teachers succeed at implementing teaching strategies that enhance students’ critical thinking skills (Miri et al., 2007). However, the laboratory is not the only hands-on activity that can be used in the classroom. Effective science teaching approaches should include for the science several types of practical activities (or Making). Some examples of Making could be found in the use of ICT in the classroom, in the production of scale models and in the organization of trips with formative aims. Making may augment other forms of learning activities, as the traditional transmission lessons; or it may provide a context for assessing students’ understanding of the scientific practices, such as experimental design in laboratory activity (Bevan, 2017).

Constructivism suggests that people’s actions are influenced by ideas and theories constructed earlier based on everyday experiences, and this applies to both students and teachers. Thus, teachers’ epistemological beliefs have been hypothesized as a central variable influencing teaching approach (Hewson and Hewson, 1987; Tsai, 2002; Kang and Wallace, 2005). There are several aspects of teachers’ beliefs that might influence their teaching approach: beliefs about the nature of science, beliefs about how to teach science, and beliefs about how students learn science. A previous study conducted on teachers’ epistemological beliefs suggested that most teachers hold a traditional view of teaching and science according to which science is best taught by transferring knowledge, giving clear and firm concepts to students, and presenting scientific truths and facts (Tsai, 2002).

Hestenes et al. (1992) assembled a large database of test results from a standardized test on force (FCI, Force Concept Inventory), demonstrating two trends: traditional teaching methods, based on lecture and homework, did not lead to substantial improvements in learning the laws regarding force and the motion as measured by the FCI, and interactive engagement generated much more substantial learning gains on the FCI. Many studies have focused on identifying aspects of the teaching approach that influence students’ conceptual understanding of physics, but focusing mostly on epistemological beliefs (e.g., Lederman et al., 2002) or testing the efficacy of instructional components (e.g., use of laboratory, Kang and Wallace, 2005), rather than focusing on the overall teaching approach, including epistemological approach, activities implemented, views on the nature of learning, use of laboratory or classroom discussion, and the like.

### This Study

In this study, we investigated the association between teaching approach and high school students’ conceptual understanding of a physical topic (i.e., force and motion). Rather than testing the efficacy of a research-designed intervention, we wanted to analyze difference between teaching practices influenced by real-life teaching approaches. We also included science-related beliefs and critical thinking as control variables for two main reasons: they are associated with conceptual understanding of physics, thus representing a potentially confounding variable; and a growth in these skills is desirable and an expected effect of a science course. We applied a mixed-method with sequential design (Johnson and Onwuegbuzie, 2004; Creswell and Plano Clark, 2011), according to which a research question is explored with a quantitative and a qualitative method. Data streams are intermixed to benefit from the strengths of both approaches. High school students’ conceptual knowledge of physics (i.e., force and motion), science-related beliefs, and critical thinking skills were assessed twice, at the beginning and at the end of the school year (quantitative approach). Physics teachers were interviewed to find similarities and differences in teaching methods (qualitative approach). Finally, the influence of the teaching method on students’ growth from the beginning to the end of the school year was investigated (intermixture between quantitative and qualitative approach). We expected to identify two main approaches of teaching physics, one more content-centred and one more student-centered, and we expected the latter approach to foster a higher increase in students’ conceptual understanding of physics, science-related skills and critical thinking skills than the former one.

## Materials and Methods

### Participants

The participants of this study were 84 high school students, enrolled in Grade 10 (Age = 15.80 ± 0.43; 59 males and 25 females). Students came from four different classes, from two different high schools located in a mid-size city in Central Italy. All students spoke Italian as their mother-tongue language. At the time of the study, no participant was diagnosed with a physical or mental disability, was included in a diagnostic process, or identified by the teachers as having special educational needs, thus all participants could be defined as typically developing. The two schools were located in areas characterized by a middle-high socio-economic level. The participating schools were not following any specific program to empower relevant variables for this study and adhered to the national curriculum. This study was carried out in accordance with the recommendations of AIP (*Associazione Italiana Psicologi*, Italian Association of Psychologists) and of the University of Florence, Italy. Ethics approval was not required at the time the research was conducted by the University of Florence. Participants’ parents subjects gave written informed consent in accordance with the Declaration of Helsinki (World Medical Association, 2013).

### Procedure and Research Design

All physics teachers working in the territory were contacted for a meeting with the researchers, in which the aims of the study were explained. Teachers were eligible to participate in the study if they had a minimum of five years’ teaching experience; were teaching Grade 10 at the time of the study; were teaching the concept of force and motion; and were not following any experimentation or specific program at the time of the study, nor had their grade 10 students followed any specific program the year before (grade 9, which in Italy is the first year of high school). Five teachers were considered eligible and expressed interest in participating in the study. During the research, one teacher had to take leave for personal reasons, and consequently was excluded from the data analysis.

At the beginning of the school year, in October, we measured students’ performances in conceptual understanding of physics, science-related beliefs, and critical thinking. In the middle of the school year (i.e., March), after the topic of force and motion had already been introduced and concluded in each class, participating teachers were interviewed (interviews were audio-recorded and transcribed for qualitative analysis). At the end of the school year, in May, students’ performances in conceptual understanding of physics, science-related beliefs, and critical thinking were measured again. All steps were conducted by a researcher trained by the first and second author of the study.

As a result of the analysis of teachers’ interviews, two groups were identified, one applying a student-centered approach (two teachers, 39 students) and one applying a content-centered approach (two teachers, 45 students). All teachers used the laboratory, lectures, and classroom discussion several times during the last school year. However, the order of these teaching components differed, as we will discuss further on in the manuscript. For instance, while the two student-centered teachers claimed to use the laboratory as a starting point of a teaching unit, the content-centered teachers took students to the laboratory after lectures, to apply the theoretical principles addressed in. Another source of differences between the two groups was the use of hands-on activities other than the laboratory. Only the student-centered teachers claimed to use alternative hands-on activities to replace the laboratory, such as reading of original texts written by past scientists or field-trips. For all the other teaching components (type of exams, material available in the laboratories, syllabus, time allotted to a teaching unit within the course) the four classrooms were equivalent.

### Measures

#### Conceptual Understanding of Physics

This variable was measured through the Force and Motion Conceptual Evaluation (FMCE, Thornton and Sokoloff, 1998), a multiple-choice test of students’ conceptual understanding of Newton’s Laws of Motion. Scores on the FMCE are strongly related to students’ score in the force concept inventory (FCI, Hestenes et al., 1992), a widely applied test measuring students’ understanding of one-dimensional kinematics and Newton’s laws; two-dimensional motion with constant acceleration; impulsive forces; vector sums; cancellation of forces; and identification of forces. In this study, we opted for the FMCE to measure students’ conceptual understanding of physics, as it provides a detailed measure of their understanding of one-dimensional forces and motion (Thornton et al., 2009), the unit of study chosen as a reference to ask teachers about their teaching method. Previous studies had used the FMCE with high school and college students, and proved its validity and reliability (Ramlo, 2008). The FMCE consists of 43 questions, and multiple choices range from five to nine answers. Overall, questions aim at assessing whether students are able to adopt a Newtonian framework or, conversely, rely on everyday experience-based conceptions. Questions use a natural language and graphical representations (e.g., [Questions] 8–10 refer to a toy car which is given a quick push so that it rolls up an inclined ramp. After it is released, it rolls up, reaches its highest point and rolls back down again. *Friction is so small that it can be ignored*.^1^ The text is followed by a graphical representation of a car on an inclined ramp facing downwards (Thornton and Sokoloff, 1998, p. 347). The test was translated into Italian by a bilingual researcher, and back-translated by another bilingual researcher. The two versions were compared and no significant differences were found. The Italian version was also expert-validated by two Physics teachers with more than 20 years of experience in teaching high-school students. Minor revisions in wording were suggested, with no semantically or conceptually significant departures from the original version. Students’ scores could range between 0 and 43, and reliability scores were ω^2^ = 0.72 at the pre-test and ω = 0.88 at the post-test.

#### Science-Related Beliefs

Students’ science-related beliefs were assessed through a self-report instrument (developed by Conley et al., 2004; Italian version by Mason et al., 2010, 2013). The instrument taps four dimensions of science-related epistemological beliefs: source (e.g., “Whatever the teacher says in science class is true”), certainty (e.g., “All questions in science have one right answer”), development (e.g., “Sometimes scientists change their minds about what is true in science”), and justification (e.g., “Good answers are based on evidence from many different experiments”) through 26 items on a 5-point Likert scale (from strongly disagree to strongly agree). The instrument was originally developed for elementary-school students (Conley et al., 2004), but has also been successfully implemented with high-school students, and proved to be valid and reliable (Tsai et al., 2011). As the main focus of the present study was not epistemological beliefs, we calculated a total score to assess students’ overall sophistication in science-related beliefs. Students’ scores could range between 26 and 130, and reliability scores were α = 0.75 at the pre-test and α = 0.83 at the post-test.

#### Critical Thinking

Students’ critical thinking was assessed through the Cornell Critical Thinking Test – Level X (CCTT; Millman et al., 2005). The test includes 71 multiple-choice items (three alternatives) and assesses the following skills: hypothesis-testing skills, credibility of source and observation skills, deduction skills, and assumption identification skills. The test is delivered in a narrative context, in which students follow the events of a group of explorers that landed on a planet to find out what happened to the first group of explorers. An example of item was as follows: “You are given two reports, you have to read them both and decide whether one of them is more credible than the other. (A) The mechanic analyses the rivers around the village and reports, the water is not drinkable; (B) the medical officer says, we still cannot tell whether the water is drinkable; (C) A and B are equally credible.” In this case, the right answer is B, since the medical officer should have more expertise on drinkable water than the mechanic has). The test was translated into Italian by a bilingual researcher and back-translated by another bilingual researcher. The two versions were compared, and no significant differences were found. The Italian version was also expert-validated by two teachers with more than 20 years of experience in teaching to high school students. Students’ scores could range between 0 and 70, and reliability scores were α = 0.76 at the pre-test and α = 0.82 at the post-test.

#### Semi-Structured Interview

Teachers were interviewed at a time agreed with them by a trained researcher, with no prior relationship with the teachers. At the time, when the interviews were conducted during the school-year, thus before post-test. Thus, at the time in which they were conducted, students’ gains in target variables were still unknown. Interview duration ranged between 45 min and 1 h. Interviews were audio-recorded and transcribed. The semi-structured interview included three sections: teaching experience and program, teaching method, and epistemological beliefs (see Supplementary Material for the full semi-structured interview).

In the first section, we asked questions to establish the teacher’s expertise (e.g., “how long have you been teaching for?”) and to establish equivalence in the Physics program delivered during the school year (“What was the Physics program this year, and in specific what topic related to force and motion did you discuss with the students?”). We used the first part of the semi-structured interview in order to collect objective data about the teaching practices implemented by the participating teachers.

The second section aimed at identifying teaching approaches, and questions were derived from Kang and Wallace’s (2005) study. Teachers were invited to think about how they taught physics in general, and the topic of force and motion in specific, and to describe a typical lesson. Then teachers were asked questions on the use of laboratory (e.g., “What roles do you believe lab activities play in your teaching?”); the use of group work and individual activities, and how often they used discussion to stimulate learning. Finally, teachers were asked which technique was more effective and which one was less effective in promoting conceptual understanding.

The last section aimed at investigating teachers’ epistemological beliefs of science, teaching science and learning science. Questions about beliefs of science [e.g., “After scientists have developed a scientific theory (e.g., atomic theory, evolution theory), does the theory ever change?”] were derived from Lederman et al.’s study (2002), whereas questions about beliefs of teaching (e.g., “Could you describe what an ideal science teaching environment would look like?”) and learning science (e.g., “What do you think about the responsibilities of students when learning science?”) were derived from Tsai’s study (2002).

Transcripts of teachers’ semi-structured interviews were investigated through thematic analysis, “a method for identifying, analyzing, and reporting patterns (themes) within data” (Braun and Clarke, 2006, p. 6). Thematic analysis allows one to search for themes across the entire data set, rather than within a data item (Braun and Clarke, 2006). In this study, we searched themes across interviews, rather than, for example, counting the frequency of specific aspects within each interview. Transcripts were analyzed after the post-test stage, but before students’ score were analyzed, thus coders were blind towards post-test group differences.

## Results

Descriptive results are presented in Table 1. An analysis of students’ conceptual understanding of physics reveals very low scores at the pre-test, and an increase at the post-test, although students’ performances are still far from full mastery of Newtonian perspective.

**Table 1:** Descriptive results (minimum and maximum scores, mean, and standard deviation) for the total sample (*n* = 84) and divided by group (student-centered *N* = 39, teacher-centered *N* = 45), and correlation among variables for the total group.

		Total					GCA		CCA							
		*N*	Min	Max	*M*	SD	*M*	SD	*M*	SD	1	2	3	4	5	6
1	Beliefs – 1	84	60	98	77.38	7.24	77.08	8.04	77.63	6.56	1					
2	Critical thinking – 1	84	33	56	46.15	5.24	45.26	5.30	46.85	5.15	0.32**	1				
3	Physics – 1	84	2	23	9.29	4.66	9.35	4.66	9.30	4.84	0.11	0.30**	1			
4	Beliefs – 2	84	86	125	108.03	8.43	107.84	10.16	108.17	7.02	0.57**	0.27*	0.05	1		
5	Critical thinking – 2	84	22	62	46.07	7.08	44.74	5.71	47.00	7.84	0.11	0.41**	0.19	0.35**	1	
6	Physics – 2	84	2	38	12.33	7.24	14.91	7.41	10.70	6.82	0.09	0.24*	0.41**	0.16	0.10	1

*^∗∗^*p* < 0.01; ^∗^*p* < 0.05. GCA, guided-constructivism approach; CCA, content-centered approach*.

The analysis of correlational scores showed that initial levels of critical thinking were associated with conceptual understanding of physics as assessed at both time points. Science-related beliefs were associated with critical thinking skills at both time points, but they were not associated with conceptual understanding of physics. Each variable at the post-test was associated with the initial performance as assessed at the pre-test.

### Teaching Approach

The qualitative analysis of teachers’ interviews, carried out with the method of thematic analysis, revealed the existence of two main teaching approaches: one defined as “guided-constructivism approach” (GCA) characterized by a focus on students’ conceptualization and guided by teachers; and another one defined as “content-centered approach” (CCA) and characterized by a traditional teaching approach (see Table 2 for a comparative chart). Interestingly, the two teaching methods differed for characteristics of the teaching method used, but not for educators’ science-related beliefs. Indeed, from the analysis of the questions derived from the teachers’ epistemological beliefs of science (Lederman et al., 2002), no substantial difference emerged, and teachers expressed similar views on the nature of science. Teachers’ epistemological beliefs are a central variable influencing teaching approach (Hewson and Hewson, 1987; Tsai, 2002; Kang and Wallace, 2005), and a potentially confounding variable in this study. Use of laboratory, classroom discussion, attribution role to students, and teachers may depend on their beliefs about the nature of science, but in this study all teachers are considered equivalent. Moreover, teachers’ sophisticated epistemological beliefs are probably associated to their students’ growth in science-related beliefs.

**Table 2:** A comparison chart between significant dimensions derived by interviews to GCA and CCA teachers.

Dimension	Guided-constructivism approach (GCA)	Content-centered approach (CCA)
Teachers’ epistemological beliefs of science	Sophisticated beliefs	Sophisticated beliefs
Goal of instruction	Explaining phenomena that students can observe in everyday life	Transmitting the skills necessary for a theoretical and abstract understanding of physical phenomena
Start of teaching unit	Laboratory (and students’ direct experience) as the start of the teaching unit	Lecture as the start of the teaching unit
Laboratory	It is the ideal place to make students dissatisfied with their naive theories and needs to be coupled with progressive discussion. Actually, they go beyond the distinction between laboratory and classroom teaching, with these two becoming mere physical places, rather than methods. One could have a laboratory in a classroom, or lecture in the laboratory.	It is a place suitable for group work, in which students can work together on experiments.
Students’ pre-instructional conceptions	Pre-instructional conceptions made by the students represent the starting point of a lesson	Instruction should aim at correcting students’ pre-instructional conceptions
Students’ participation in the classroom discourse	Laboratory is not always effective in eliciting students’ conceptions about physics topics, and field-trips as well as videos or reading of original writings by past scientists should be used to relate physics concepts to real life experiences.	CCA teachers simply rely on the laboratory and do not implement alternative participatory activities in their teaching of physics.
Students’ role	Students should have an active role to students in the deconstruction of naive schemes and conceptual understanding of physics concepts	Students should be motivated to learn.
Teacher’s role and guidance	Lectures, laboratory activity and classroom discussion should be guided by the teacher. Overall, students need to be guided throughout all the steps of science learning. Teachers need to be aware of students’ pre-instructional conceptions and guide them through scientifically valid conceptions.	Teachers should explain the theory and set up experiments for students

Conversely, from the qualitative analysis of the questions on the teaching approach (derived from Tsai, 2002; Kang and Wallace, 2005), significant differences emerged.

Although all four teachers showed the use of some typical constructivist teaching techniques, the two GCA teachers claimed to have a more substantial focus on the conceptual construction of concrete meanings of physics. In other words, the teachers aimed at explaining phenomena that students can observe in everyday life. The two CCA teachers stated that in the classroom they aimed at transmitting the skills necessary for a theoretical and abstract understanding of physical phenomena, without explicitly mentioning the importance for the students to become able to understand the real phenomena (see Q1 in Table 3). Importantly, no teacher mentioned the explicit teaching of higher-order skills, which is probably associated to the lack of growth in critical thinking skills over the school year in the students of our sample (Miri et al., 2007).

**Table 3:** Excerpts from participating teachers’ interviews representing differences in teaching approach.

Questions	GCA teacher #1	GCA teacher #2	CCA teacher #1	CCA teacher #2
*(Q1) How would you describe your teaching approach in Physics?*	Contextualizing the concepts and showing how the theories have developed in history to understand everyday aspects of life.	Showing students how things work through knowledge of Physics concepts.	I try to get students accustomed to a theoretical dissertation a bit abstract	General dissertations, without being too specific
*(Q2) What is the ideal teaching approach to Physics*	Having the class in the laboratory, because facts and phenomena, the things you observe and perceive should be the starting point; because if we use book pages or notes as the starting point, students simply repeat the lesson and they do not even know what they are talking about.	What I noticed is that if you match a theoretical class with a practical one, learning is more enduring. The ideal method is to be in the laboratory, a laboratory fully equipped, rather than having a whiteboard class, and start a topic from a practical situation that students can perceive.	Matching individual studying with a theoretical discussion in the classroom	Lecture and subsequent discussion in classroom aiming at correcting errors
*(Q3) When is classroom discussion productive*	It is productive when we try to emphasize the most important claims, but also the incorrect ones. We actually linger on the incorrect claims, we make them emerge and become more explicit. Large part of classroom discussion consists of starting from making students aware of their common-sense beliefs, and transforming them into scientific conceptualization.	It is productive when there is participation, including also the students who generally are more resistant to participate. It is productive when I ask some questions and students try to answer them, even if their answers are wrong I still consider the discussion productive, as it opens up an issue, and represents a starting point.	It is productive when students take turns in speaking and are all involved.	In a classroom discussion, I am the one asking questions. I let students answer them, stressing the right one, and correcting the wrong ones.
*(Q4) What activities do you consider most successful in your teaching approach to elicit students’ understanding of physics of force and motion?*	Reading of original texts written by past scientists, collective discussion of everyday physics-related aspects of life, images or videos.	Sometimes I take students out of the classroom into contexts in which physics concepts can be observed. Once I took them to D’Azeglio square to examine the oscillating motion of swings. Once, a student shot a video of angular motion (with him sitting on a stool) and we watched it and discussed it in class.	I do not implement any particular activity with the topic of force. With other topics we might do some field-trips, but not very often.	99% of the time I teach through lectures.
*(Q5) What is the students’ role in science learning?*	Students should synthesize what we discuss together in the classroom. They should elaborate on the topics and reach a deep understanding of concepts. A big challenge is to remove common-sense beliefs about force and motion, although they are persistent. I let the knowledge be constructed by them; I think that learning is effective when they reach a conclusion on their own. Once they reach a conclusion and revise a common-sense belief, the teacher’s job is to underline what has happened and make sure that students are aware about the conceptual change that took place.	Students’ responsibility is to elaborate on what they have empirically experienced, regardless of whether they experienced it incidentally or in school. The teachers should promote this type of reflection.	Students need to feel involved in the classroom	Students should find the motivation to get engaged.
*(Q6) What is the teachers’ role in science learning?*	An important task is to remove students’ common-sense conceptions, and transform them into scientifically correct Newtonian conceptions. In the laboratory, it is important that the teacher stimulates certain reflections. Sometimes I conduct the experiment, or I have the students conduct it in small groups, and I go around to be sure that they are reflecting During classroom discussion, the teacher should capture the most significant aspects, and synthesize them. My role is that of a guide. Students can briefly discuss among themselves, but they should also be led towards a collective discussion, which needs to be guided to be effective.	The teacher needs to put the students in condition to practice concepts; the teacher should not just talk about them. The laboratory activity means asking a question to students. Students need to be guided as they should always be aware of what they are doing and why they are doing certain things. My role is to communicate what we do and the main problem we are inquiring. I participate in their work, but I also observe, overall, they need to be masters of the activity.	My main responsibility is that they feel involved. I ask them to stop me when they do not understand something. Then, they need to proceed on their own, and elaborate knowledge rather than learning concepts by heart.	Three functions, (1) explaining what they need to do; (2) guide them through theory; and (3) be sure that all the safety norms in the laboratory are respected

Most teachers would say that the laboratory is important in science teaching, but they might differ in the role attributed to it in their lesson plan. Moreover, their actual use of laboratory in their teaching practices might depend on availability of instruments and thus, change from school to school. Therefore, we asked teachers to describe their ideal teaching approach. As a consequence of this focus on concrete concepts, GCA teachers affirmed that the ideal teaching method should be to start from the laboratory and from students’ direct experience of the physical phenomena that are going to be discussed in classroom. CCA teachers consider the laboratory as an important aspect of physics teaching too, but they believe that the starting point of teaching should be the lecture. Whereas students’ pre-instructional concepts are often viewed by teachers as obstacles to science learning, GCA teachers consider them as resources to successfully begin a teaching unit (Larkin, 2012). CCA teachers see the laboratory as a place suitable for group work, whereas GCA teachers believe that the laboratory is the ideal place to make students dissatisfied with their naive theories, thus provoking in them the cognitive dissonance necessary to motivate them towards change and learning of correct concepts on the phenomena of physics (see Q2 in Table 3). GCA teachers organize the laboratory activity in brief, qualitative observations, always fostering individual reflection and collective discussion, acknowledging the importance of integrating inquiry-based activities with classroom talk (Mortimer and Scott, 2003). In this way, they go beyond the distinction between laboratory and classroom teaching, with these two becoming mere physical places, rather than methods. The laboratory, as well as the classroom discussion, are seen by constructivist teachers as moments where it is possible to start from students’ mistakes to help them construct correct theories on empirical phenomena. In this perspective, GCA teachers, unlike CCA ones, believe that the mistakes made by the students represent the starting point of a lesson. Pre-instructional concepts, should be valorized, rather than corrected, stimulating in the student the cognitive reasoning preliminary to conceptual change and learning (see Q3 in Table 3). Of notice, students derive their pre-instructional concepts from everyday experiences and will not revise them if simply exposed to new theories, unless they are provided with reflective experiences (Boddy et al., 2003).

GCA teachers also use several means to foster students’ participation in the classroom discourse on science. Laboratory is not always effective in eliciting students’ conceptions about physics topics, and field-trips as well as videos or reading of original writings by past scientists (who sometimes struggled with the same pre-instructional conceptions our students hold) might support teachers in this step (see Q4 in Table 3). Most of the time, CCA teachers simply rely on the laboratory and do not implement alternative participatory activities in their teaching of physics, although prior studies have shown that physics classrooms based on progressive discourse greatly increase students’ conceptual understanding of physics more than content-centered classrooms do (Bigozzi et al., 2014).

Another important source of differences between the two teaching approaches identified in this study lies in what role teachers attribute to students in science learning. Whereas CCA teachers attribute a motivational role to students, GCA teachers emphasize the importance of attributing an active role to students in the deconstruction of naive schemes and conceptual understanding of physics concepts (see Q5 in Table 3).

GCA teachers are aware that students need to be guided throughout all the steps of science learning. Teachers need to be aware of students’ pre-instructional conceptions and guide them through scientifically valid conceptions. As such, also the laboratory activity and classroom discussion should be guided by the teacher (see Q6 in Table 3). Past studies have shown that constructivist approaches with minimally guidance by the teacher are not effective in promoting conceptual understanding of scientific concepts (Kirschner et al., 2006; Zhang, 2018).

### Groups’ Equivalency at the Beginning of the School Year

To determine the equivalence between groups at the beginning of the school year, we conducted a series of *t*-tests for independent samples, with group as independent variable and pre-test scores in science-related beliefs, critical thinking and conceptual understanding of physics as dependent variables. We used the two one-sided tests (TOST) procedure for testing equivalence (Lakens, 2017). Whereas traditional t-tests for independent samples allow to refuse the null hypothesis, the TOST procedure allows to verify equivalence between means. No significant differences emerged, so we could conclude that the two groups were equivalent (see Table 4).

**Table 4:** Pre-test differences between groups: Results from TOST Independent Samples *t*-test.

	*t*	*Df*	*p*	95% LCI	95% UCI
Beliefs – 1	-0.20	74.9	0.84	-3.51	2.76
Critical Thinking – 1	-1.28	63.7	0.21	-3.68	0.50
Physics – 1^+^	0.05	69.4	0.73	-0.07	0.10

*^+^Variable normalized through monotonic transformation*.

### Effect of Teaching Approach on Students’ Gains

Research hypotheses were explored through a generalized linear model for complex samples (complex-samples GLM) conducted with the software IBM SPSS version 19. Complex-samples GLM allows to control the effect of data nested within clusters (in our case, classrooms), and thus to test group differences with adjustment for clustering by classrooms (Aerts et al., 2002). Educational studies have often do deal with clustered data. Clustered data arise when the data from the whole study can be classified into a number of different groups, referred to as clusters, and observations within a cluster are more alike than observations from different clusters (Galbraith et al., 2010). Each cluster contains multiple observations, giving the data a “nested” or “hierarchical” structure, with individual observations (i.e., students) nested within the cluster (i.e., classrooms). Modeling approaches are particularly useful when there are other covariates that need to be included in the analysis (Galbraith et al., 2010), such as in the case of the present study.

Classroom was included as cluster variable to account for random effects. Group was included as factor to analyze differences between teaching approaches in post-test scores. Outcome variables were post-test scores in science-related beliefs, critical thinking, and conceptual understanding of physics. Pre-test scores in science-related beliefs, critical thinking, and conceptual understanding of physics as covariates, to account for initial differences.

The group variable explained post-test performances in conceptual understanding of, but not in science-related beliefs. Post-test scores in science-related beliefs, critical thinking, and conceptual understanding of physics were all associated to their respective pre-test scores. Post-test scores in conceptual understanding of physics were also associated to pre-test scores in critical thinking (see Table 5).

**Table 5:** Results from the complex samples GLM.

Dependent variables	Parameters	*df*	Wald’s *F*	*p*
Physics – 2^+^ [*R*^2^ = 0.39]	Group	1, 3	15.50	0.03
	Physics – 1^+^	1, 3	7.95	0.07
	Beliefs – 1	1, 3	0.35	0.60
	Critical thinking – 1	1, 3	8.72	0.06
Beliefs – 2 [*R*^2^ = 0.43]	Group	1, 3	8.40	0.06
	Physics – 1^+^	1, 3	0.15	0.73
	Beliefs – 1	1, 3	15.61	0.03
	Critical thinking – 1	1, 3	6.67	0.08
Critical thinking – 2 [*R*^2^ = 0.32]	Group	1, 3	0.31	0.62
	Physics – 1^+^	1, 3	1.25	0.35
	Beliefs – 1	1, 3	0.17	0.71
	Critical thinking – 1	1, 3	18.91	0.02

*^+^Variable normalized through monotonic transformation*.

Overall, both teaching approaches are effective in promoting growth in science-related beliefs, probably because of teachers’ sophisticated epistemological beliefs. Neither of the two teaching approaches are effective in promoting students’ critical thinking skills, probably because they fail at embedding explicit teaching of higher order skills in their teaching practices (Miri et al., 2007). Whereas both teaching approaches may be effective in promoting a learning of theoretical principles and laws, the GCA approach is more successful in promoting conceptual understanding of physics concepts.

## Discussion

This study contributes to our understanding of teaching approaches to physics in high school, and how they are associated with students’ conceptual learning of force and motion. To evaluate the teaching method and in order to understand which characteristics of it could predict students’ performance, we implemented a qualitative thematic analysis of the teachers’ interviews. The semi-structured interview investigated teachers’ teaching approach about physics in general and the topic of force and motion in specific (i.e., use discussion, laboratory, individual and group work in their teaching practices, their epistemological beliefs about science, and their epistemological beliefs about teaching science. The questions were derived from past studies (Lederman et al., 2002; Tsai, 2002; Kang and Wallace, 2005).

Past studies have suggested that teachers’ epistemological beliefs about science play an important role in their teaching practices in the classroom (Hewson and Hewson, 1987; Lederman et al., 2002; Tsai, 2002; Kang and Wallace, 2005), but in our study teachers held similar views on the nature of science, allowing us to consider them equivalent in epistemological beliefs on science, and focus our analysis on the teaching approach only. Teachers might hold sophisticated beliefs about the nature of science, but these do not automatically transfer to their practices (Yoon and Kim, 2016). Moreover, teachers reported similar teaching practices, which are generally associated to general principles of constructivism (use of laboratory, importance of discussion, assigning an active role to students, and the like). For example, all teachers affirm that when they teach physics to students they start to explain to them the real events that each student knows. In other words, all teachers explained the physics concepts starting from students’ experiences, and this is an important aspect in the constructivist method (Mortimer and Scott, 2003; Bigozzi et al., 2014). Thus, on surface, all teachers believed that they were teaching according to constructivist principles. Differences emerged when teachers were asked about their practices when teaching about force and motion (Mansour, 2009), that is, when their teaching approach was inquired more in depth. The thematic analysis revealed the presence of two main teaching approaches, one defined as guided-constructivism approach, and the other one as content-centered approach. The two GCA teachers attributed a specific role to the laboratory, an integrated component in the teaching practice in which students can have experience of their own beliefs, rather than using it as “a frill” (Tobin, 1986; Kang and Wallace, 2005). In this study, GCA teachers assigned a seminal role to the laboratory, as it gives rise to the whole teaching module. Moreover, the laboratory setting allows teachers to guide also the moment in which students become aware of their own and each other’s pre-instructional conceptions. Some teachers interpret constructivist teaching as unguided teaching, but this approach has been demonstrated to be ineffective (Kirschner et al., 2006). Rather than having students discover laws and principles by improvising as scientists (van Joolingen et al., 2005), they should be guided in each step of the inquiry, and supported to become aware of their own conceptions and provided with a reflective experience (e.g., experiment in the laboratory, field-trip, video, historical readings, and the like) on the perceived phenomena (Boddy et al., 2003). For instance, a laboratory activity should also be integrated with classroom talk (Bereiter, 1994; Mortimer and Scott, 2003). The laboratory activity should produce knowledge in students. Laboratory activity and classroom lectures should not be considered as distinct moments. In choosing which experiment to engage students with, the teacher needs to choose one that might create cognitive dissonance in the students, make them ask questions, and foster desire of knowing. GCA teachers asked students a disposition towards conceptual change in a guided environment, rather than being the only agent of such a conceptual change. CCA teachers tend to value students’ performance in terms of conformity to the criteria of the discipline, and consider the evaluation and correction of the learner’s conceptualization as the main teacher’s task (Mansour, 2009).

The analysis of post-test scores revealed that the GCA teachers’ students outperformed the CCA teachers’ students in conceptual understanding of force and motion at the end of the school year, after checking on the effects of conceptual understanding of force and motion, critical thinking and science-related beliefs at the beginning of the school-year. No group differences were found for post-test science-related beliefs or critical thinking. An analysis of descriptive scores shows that critical thinking does not increase from pre-test to post-test, whereas science-related beliefs appear to improve in both groups. Thus, the reason why the teaching approach does not influence these variables might differ. Critical thinking might not improve as neither of these two approaches explicitly targets it. One might expect an improvement in critical thinking skills as an ancillary effect of GCA, but teachers might need to increase guidance in this direction. For instance, exposing students to the original writings of famous physicists might improve their conceptual understanding of physics, but unless these writings are compared with non-authoritative writings, students do not reflect on the differences between scientists’ approach to a problem versus laypeople’s approach to it, and do not practice (or improve) observation and credibility of sources skills. For what concerns science-related beliefs, they appear to improve in both groups over the school year, so the two teaching approaches might be equivalent in their efficacy.

In conclusion, secondary school teaching can be meaningful if a balance between experimentation and observation, historical contextualization, use of videos and simulation, is achieved. Of course, such a balance must take into consideration the school resources. The simultaneous and balanced use of all these methodological instruments allow one to create classrooms based on scientific knowledge construction, within which textbooks are just one (and not the main) of the several learning aids. Rather than transmission of knowledge by the teacher, physics teaching should be characterized as a shared construction of knowledge, which is the result of the collective synthesis of a learning process that has been organized and guided by the teacher.

### Limitations and Directions for Future Research

When interpreting the findings of the current study, some limitations should be taken into account. First, the focus of this study was on the teachers’ perception of their own teaching approach, and to what extent these differences are accountable for variance in students’ conceptual understanding of physics. However, prior studies have emphasized the existence of a gap between what teachers think constructivism is, and the way in which they actually teach (Mortimer and Scott, 2003). Thus, future research should complement the research design of this study by including classroom observation too, targeting all the components of the teaching approach (lecture, laboratory, classroom discussion, group work, field-trips, and the like).

Second, the conclusions that we are able to draw on the influence of the teaching approach on students’ conceptual understanding of physics is limited to the topic of force and motion. The topic was chosen as students generally present several pre-instructional conceptions about it, and struggle to think in a Newtonian way even after being exposed to a Physics course. Other topics might impose different affordances to the learning context. Students might have fewer pre-instructional conceptions about phenomena that are rare in everyday life, or certain topic might be more difficult to observe and be connected in a clear way to concrete situations.

Finally, in the present study we aimed at controlling as many confounding variables as possible (i.e., teaching experience, grade taught, program content), which restricted the pool of eligible teachers. Future studies we aim at verifying whether the results of this study apply also when the controlled variables are manipulated.

## Conclusion

Despite the limitations, the present study contributes to the literature on students’ conceptual learning of physics in several ways. It contributed to create a semi-structured interview that includes several components, all associated to students’ learning performance: beliefs about the nature of science (Lederman et al., 2002), beliefs about teaching and learning science (Tsai, 2002), and use of laboratory (not as a separate moment from the classroom lecture, but as a key moment of knowledge building when integrated with other components such as discussion and group work) in the teaching practices (Kang and Wallace, 2005). In specific, in the interview we asked questions on their ideal teaching approach and their actual teaching approach, asking them to anchor their answers to the way they had taught force and motion during the school year (Mansour, 2009).

It also contributed to our understanding of which component of the teaching approach is associated with students’ progress in physics and critical thinking skills. Several studies have investigated the influence of teachers’ beliefs about the nature of science, but in this study all teachers held equivalent views, and differed on other crucial components. Having sophisticated epistemological beliefs is a necessary but not sufficient condition to create a learning environment that fosters conceptual learning. Finally, results of the study contributed to our understanding of the role that specific components of the teaching approach should have. Simply going to the laboratory does not foster a constructivist learning in students, unless it is matched with reflection. Specifically, our results suggest that the laboratory should be a guided experience that should be offered to students at the beginning of a teaching unit, with the purpose of making them aware of the difference between their pre-instructional conceptions and the manifestation of phenomena.

## Author Contributions

LB and CT designed and conducted the study, and wrote up the manuscript. CF and PF participated in the designing of the study and in the discussion of results. FS participated in the data analysis and in the discussion of results.

## Conflict of Interest Statement

The authors declare that the research was conducted in the absence of any commercial or financial relationships that could be construed as a potential conflict of interest.

## Footnotes

1 Italics are used in the original version.

2 Reliability scores for the conceptual understanding of physics measure were calculated through McDonald’s ω, because of the differences in nature between questions in the same instruments (i.e., multiple choices ranging from five to nine answers).
