# Supplementary material for: The Influence of Teaching Approach on Students’ Conceptual Learning in Physics
Source: Front Psychol. 2018 Dec 5;9:2474. doi: 10.3389/fpsyg.2018.02474 (PMC6290030; doi:10.3389/fpsyg.2018.02474)
Supplement: Supplementary file 1 [file Table_1.docx]

Supplementary Material

The influence of teaching approach on students’ conceptual learning in Physics

Lucia Bigozzi, Christian Tarchi*, Carlo Fiorentini, Paola Falsini, Federica Stefanelli

*** Correspondence:** Corresponding Author: christian.tarchi@unifi.it

# Teachers’ semi-structured interviews

1. Teachers’ variables

1.1 How long have you been teaching for?

1.2 What was the Physics curriculum this year, specifically referring to force and motion?

1.3. How did you evaluate students’ performances with reference to force and motion?

2. Teaching approach (in general and with reference to the topic of force and motion)

*2.1 Questions about the general teaching approach*

2.1.1 How would you describe your teaching approach? What were your teaching goals this year?

2.1.2 What is the ideal approach to teach physics? What is the ideal learning environment?

2.1.3 What is the ideal way to learn physics? What are the students’ responsibilities?

*2.2 Questions about the teaching approach with reference to force and motion*

2.2 Referring to the unit about force and motion, how would you describe your teaching approach?

2.2.1 What is a typical lesson about force and motion like?

2.2.2 How did you assess students’ conceptual understanding of force and motion?

*2.3 Questions about the use of the laboratory* (adapted from Kang and Wallace, 2005)*.*

2.3.1 What do you mean by “laboratory activity”?

2.3.2 How often did you go to the laboratory during the force and motion unit?

2.3.3 What other participatory activities did you use, besides the laboratory?

2.3.4 Is there a difference between laboratory demonstration and laboratory activity?

2.3.5 What role does the laboratory play in your teaching approach?

2.3.6 What makes you decide to use the laboratory?

2.3.7 What is your role in the laboratory?

2.3.8 What is the students’ role in the laboratory?

2.3.9 Some people compare the way scientists work to the way students learn physics. Do you agree or not? Why?

*2.4 Questions about the use of other teaching elements*

2.4.1 How often did you use group work in the force and motion unit?

2.4.2 How often did you use individual work in the force and motion unit?

2.4.3 Which technique was particularly effective? Which technique was not really effective?

*2.5 Questions about classroom discussion*

2.5.1 How often did you use classroom discussion in the force and motion unit?

2.5.2 When do you consider classroom discussion as productive?

2.5.3 What is your role in a classroom discussion?

2.5.4 What is the students’ role in a classroom discussion?

3. Epistemological beliefs

*3.1 Epistemological beliefs about science*

Ten questions, derived from Views of Nature of Science Questionnaire, VNOS (Lederman et al., 2002)

*3.2 Epistemological beliefs about teaching and learning science*

Three questions, derived from Tsai's study (2002)
